# Calcineurin inhibitor blocks tolerance by suppressing donor T cell terminal exhaustion after allogeneic hematopoietic cell transplantation

**DOI:** 10.1172/JCI184332

**Published:** 2024-10-15

**Authors:** Hajime Senjo, Daigo Hashimoto, Takanori Teshima

**Affiliations:** Department of Hematology, Hokkaido University Faculty of Medicine, Sapporo, Japan.

**Keywords:** Hematology, Immunotherapy

**To the Editor:** We read with great interest the recent article by Wang et al. (1), wherein the authors demonstrated calcineurin inhibitors (CNIs) administered after experimental allogeneic hematopoietic stem cell transplantation (allo-HCT) suppressed donor T cell exhaustion while promoting the expansion of CD62L^+^CD44^+^ central memory T cells (Tcm), which contribute to the development of chronic graft-versus-host disease (cGVHD). We previously reported that CNIs inhibited terminal exhaustion of donor T cells and contributed to the development of cGVHD after allo-HCT (2). Both studies share the concept that CNIs suppress the exhaustion of donor T cells and impair tolerance induction. However, the cell types induced by CNIs and contributing to cGVHD development differ between studies: transitory exhausted T cells (transitory-Tex) in our study, but Tcm in the Wang et al. study.

In our study, single-cell RNA sequencing (scRNA-seq) on day 14 after transplantation showed that the majority of alloreactive donor T cells had completed differentiation toward terminally exhausted T cells (terminal-Tex) after allo-HCT without CNI. However, CNIs downregulated *Tox* expression and inhibited differentiation of transitory-Tex toward terminal-Tex. We identified transitory-Tex based on the expression of exhaustion markers such as *Tox*, *Pdcd1*, and *Havcr2*, along with effector molecules such as *Cx3cr1* and *Gzmb*, and their self-renewal capacity, as has been previously shown (3). In Wang et al.’s study, scRNA-seq analysis was performed on day 7, which may be too early to assess T cell exhaustion. In our study, the majority of donor T cells were CD62L^–^PD-1^+^ irrespective of CNI treatment, while Wang et al. demonstrated the expansion of CD62L^+^PD-1^lo^ Tcm in CNI-treated recipients (Figure 1, A–D). TOX expression was significantly reduced by CNIs in both studies, consistent with expansion of TOX^lo^ transitory-Tex in chronic viral infection (4). Furthermore, *Tcf7*, a marker for precursors of exhausted T cells and Tcm, was expressed only in a minor population in our study. Taken together, the data show that CNI induced transitory-Tex, not Tcm, in our study.

Although the reasons why cell types induced by CNIs differ between studies are not clear, differences in study methodologies may be associated with different results. For example, cyclosporine (CSP) was given intraperitoneally in Wang et al.’s study, but orally in ours (5).

We demonstrated that Ly6C is a reliable marker of transitory-Tex, but Wang et al. disagree with this. Wang et al. reported that under CNI treatment, there is a broad increase in Ly6C expression across various T cell subtypes. We believe that we could purify transitory-Tex cells using Ly6C, because *Ly6c2* was included in the top 10 differentially expressed genes in CNI-expanded transitory-Tex. However, when we look carefully our scRNA-seq data, we now find some *Tox^–^* cells were included in Ly6C-expressing CNI-expanded clusters (Figure 1, E and F). Although these Ly6C^+^TOX^–^ cells are not Tcm based on their lack of CD62L expression, they may represent memory-committed cells specifically induced by CNI. Importantly, adoptive transfer of these Ly6C^+^ transitory-Tex gave rise to both TOX^hi^Ly6C^lo^CX3CR1^lo^Gzmb^lo^ terminal-Tex and TOX^lo^Ly6C^hi^CX3CR1^hi^Gzmb^hi^ cells. This suggests CNI-induced Ly6C^+^ transitory-Tex had been committed to the exhaustion program before the transfer. We also showed that CNI-expanded Ly6C^+^ cells responded well to PD-1 blockade, which suggests that these cells were suppressed by PD-1–mediated inhibitory signals, but were not terminally exhausted, consistent with the characteristics of transitory-Tex.

Life-long expression of host-derived alloantigens after allo-HCT likely drives alloreactive donor T cells into exhaustion. It is possible that there are specific intermediate populations endowed with memory function while differentiating toward exhaustion, and dominant cell populations may be model and context dependent. These points should be evaluated in future studies.

## Figures and Tables

**Figure 1:**
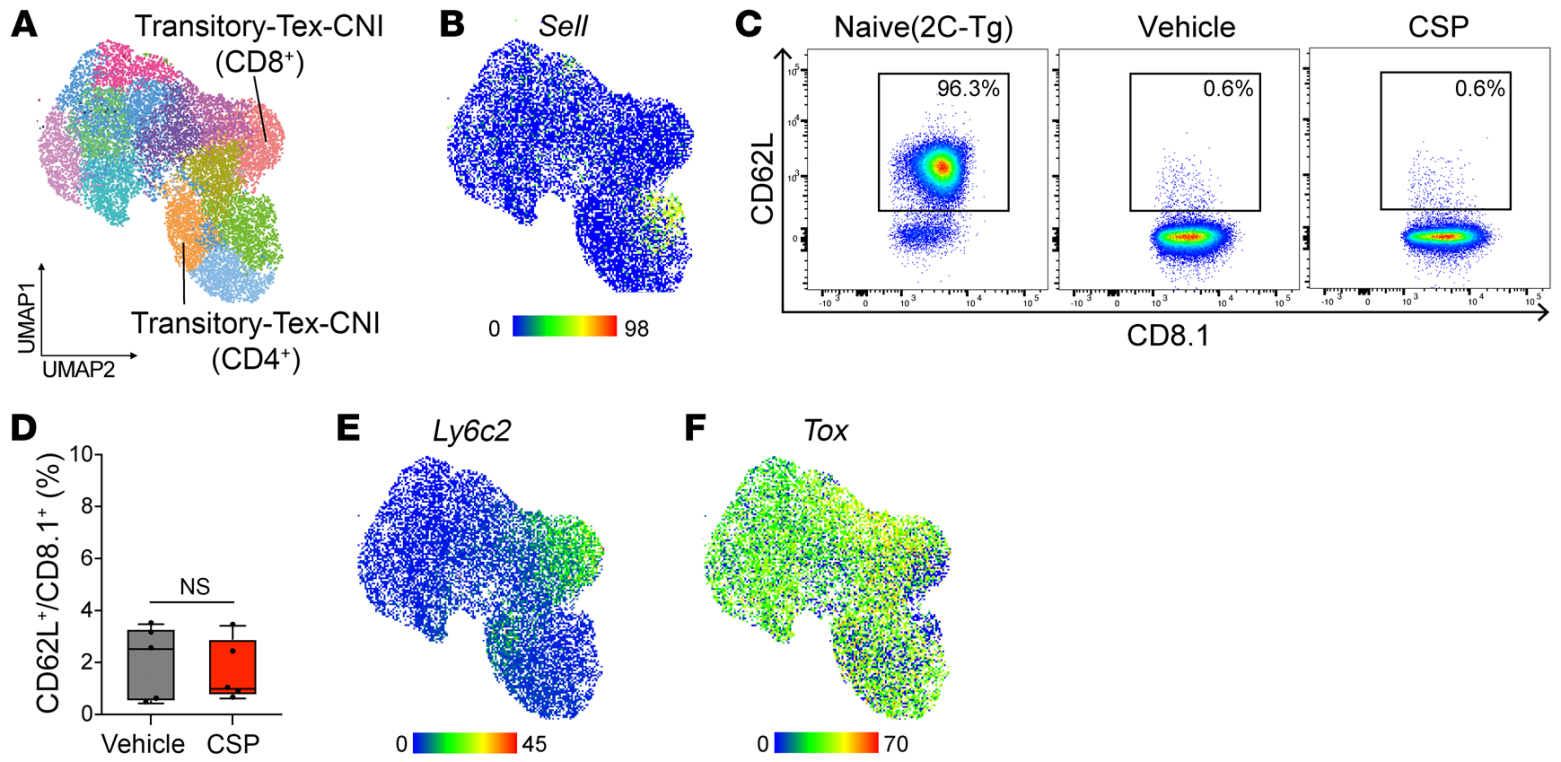
Donor T cell profile on day 14. CSP (25 mg/kg) was administered on days 0–13 after allo-HCT. (**A**, **B**, **E**, and **F**) scRNA-seq. (**C** and **D**) CD62L on 2C-Tg donor T cells (*n* = 5/group). Box-and-whisker plot shows the median (vehicle vs. CSP: 2.510 vs. 0.990; line in box), IQR (0.57–3.12 vs. 0.86–2.39; box bounds), and minimum/maximum values (0.43/3.47 vs. 0.61/3.41; whiskers). *P* > 0.99 by 2-tailed Mann-Whitney *U* test (**D**).

## Version 1 10/15/2024

Electronic publication

## References

[1] Wang Y (2024). Calcineurin inhibition rescues alloantigen-specific central memory T cell subsets that promote chronic GVHD. J Clin Invest.

[2] Senjo H (2023). Calcineurin inhibitor inhibits tolerance induction by suppressing terminal exhaustion of donor T cells after allo-HCT. Blood.

[3] Hudson WH (2019). Proliferating transitory T cells with an effector-like transcriptional signature emerge from PD-1(+) stem-like CD8(+) T cells during chronic infection. Immunity.

[4] Beltra JC (2020). Developmental relationships of four exhausted CD8^+^ T cell subsets reveals underlying transcriptional and epigenetic landscape control mechanisms. Immunity.

[5] Wassef R (1985). Pharmacokinetic profiles of cyclosporine in rats. Influence of route of administration and dosage. Transplantation.

